# Surgical Excision of Localized Gingival Overgrowth Using Laser: A Case Report

**DOI:** 10.7759/cureus.54415

**Published:** 2024-02-18

**Authors:** Ruchita T Patil, Khushboo K Durge, Shrishti S Salian, Prasad V Dhadse, Sakshi A Akolkar, Sneha C Dare

**Affiliations:** 1 Department of Periodontics and Implantology, Sharad Pawar Dental College and Hospital, Datta Meghe Institute of Higher Education and Research, Wardha, IND; 2 Department of Periodontics and Implantology, Sharad Pawar Dental College and Hospital, Datta Meghe Institute of Higher Education and Research, Wardha, IND, Wardha, IND; 3 Department of Oral Pathology and Microbiology, Sharad Pawar Dental College and Hospital, Datta Meghe Institute of Higher Education and Research, Wardha, IND

**Keywords:** enlargement, overgrowth, laser, gingiva, fibroma

## Abstract

Gingival overgrowth, localized or generalized, is one of the leading causes of poor maintenance of oral hygiene. Excision of growth using laser should be the choice of treatment because laser helps maintain a blood-free surgical site during treatment and provides more patient comfort during and after the procedure. Lasers are commonly employed in many different applications, including scaling, root planning, cavity preparation, and excision of soft tissue growths in surgery. Laser therapy offers numerous benefits over traditional methods of treatment. These benefits have led to the growing use of lasers as dental treatment options in a variety of dental fields. In this case report, we are presenting a case of excision of localized gingival growth using a laser. Postoperative healing and maintenance of oral health were satisfied after laser surgery.

## Introduction

Soft tissue enlargements of the oral cavity could be caused by inflammation or cysts, developmental anomalies, or neoplasm. Enlargement of gingival tissue can be localized or generalized. Gingival hyperplasia refers to a rise in the number of cells, and gingival hypertrophy, which refers to an increase in the size of cells, can also be used to describe gingival overgrowth or enlargement. According to histology, gingival tissue growth is associated with variable alterations in the extracellular matrix, gingival vasculature, cell size, and cell multiplication [1].

Localized soft tissue or gingival growth is commonly seen in the oral cavity. Most of these growths, including peripheral giant cell granuloma, irritational fibroma, pyogenic granuloma, and ossifying fibroma, are benign and hardly ever exhibit aggressive characteristics [2]. These lesions typically develop as a result of trauma or persistent irritation. In general, irritated or traumatic fibromas are regarded as reactionary connective tissue hyperplasia in response to trauma and irritation. They are a form of benign exophytic tumor or neoplasm of fibrous connective tissue origin. The process of chronic healing leading to a fibrous submucosal tumor is called fibroma. It involves the creation of scars and granulation tissue. Rarely, recurrences can result from repeated damage at the same location [3]. There is no chance that the lesion will become malignant. Traumatic fibroma typically presents as a smooth, hard lesion with a sessile or pedunculated base that varies in size and is hard to the touch. Its sluggish, painless growth typically occurs over months or years [4].

Gingival enlargement is a frequent disorder that affects an individual's masticatory, functional, aesthetic, and psychological health. If the cause of the gingival enlargement is readily visible, a clinical diagnosis may be made quickly. However, in order to determine the cause and create a treatment plan that works, a doctor may need to investigate the condition and find any underlying disorders, drug interactions, or physiological abnormalities. When the etiology of an illness is unknown, it is difficult to make a diagnosis and determine the prognosis [5].

The present case report reveals a benign, localized gingival overgrowth present on the mandibular anterior region between the right lateral incisor and the right lateral canine.

## Case presentation

**Figure 1:**
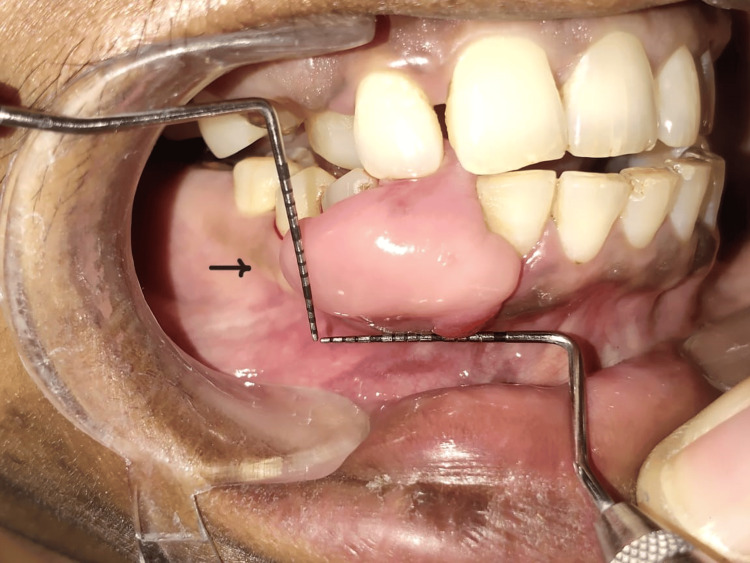
Preoperative photograph

A 41-year-old female visited the Department of Periodontics and Implantology with a chief complaint of intra-oral swelling in the lower front region for four to five months, which progressively increased in size with time (Figure 1).

There was no significant medical or dental history. The patient was referred to the Department of Oral Pathology for blood investigations, including bleeding time (BT) and clotting time (CT), as well as hemoglobin (Hb). The results of the blood investigations are shown in Table 1.

**Table 1:** Hematological findings BT - bleeding time; CT - clotting time; Hb - hemoglobin; RBS - random blood sugar

Hematology	Patient value	Normal range
Hb%	11.2 gm%	M: 12-15.5 gm%; F: 11-14.5 gm%
BT	1 min 21 sec	1-3 min
CT	2 min 30 sec	1-5 min
RBS	90 mg/dl	M: 79-160 mg/dl; F: 70-160 mg/dl

**Figure 2:**
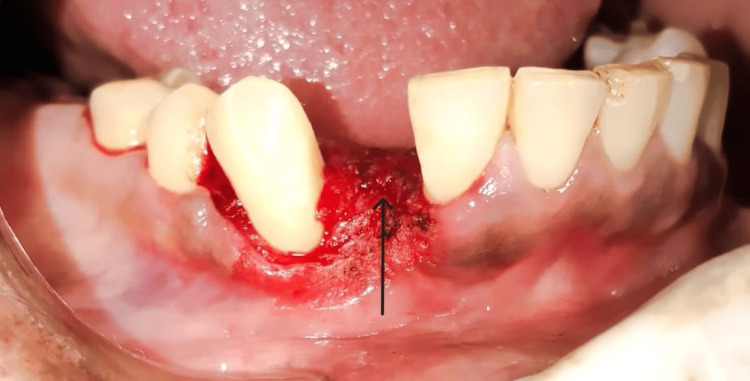
Postoperative photograph

**Figure 3:**
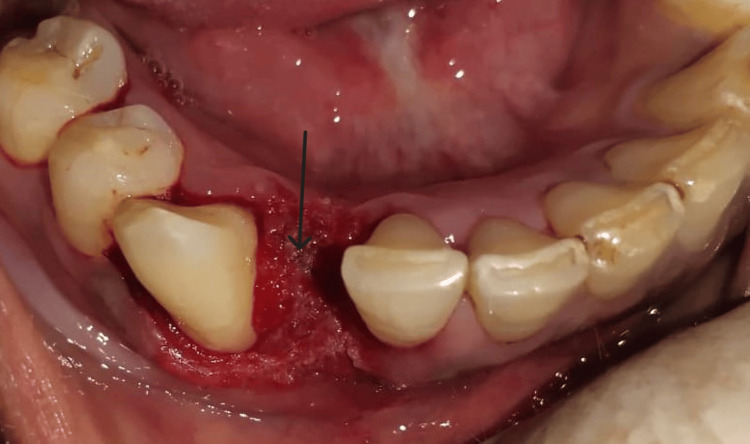
Occlusal view

After eight days, the patient was recalled for re-evaluation and surgical excision of the growth. Lignocaine (2%) with adrenaline was administered. The inferior alveolar and lingual nerve block was given to anesthetize the area. The growth was then tied using dental floss to pull it on a side for ease of the procedure. Laser (BIOLASE® Epic X Diode Laser) tip was then applied to the bottom of the tissue for excision. The tissue was excised as a whole (Figures 2-3).

A pressure pack was placed in the operated region. Postoperative instructions were given, including avoiding hot food and vigorous brushing in the operated area. Postoperative medications, including antibiotic amoxicillin (500 mg), aceclofenac (100 mg), serratiopeptidase (15 mg), and paracetamol (325 mg), were prescribed to the patient for three days.

**Figure 4:**
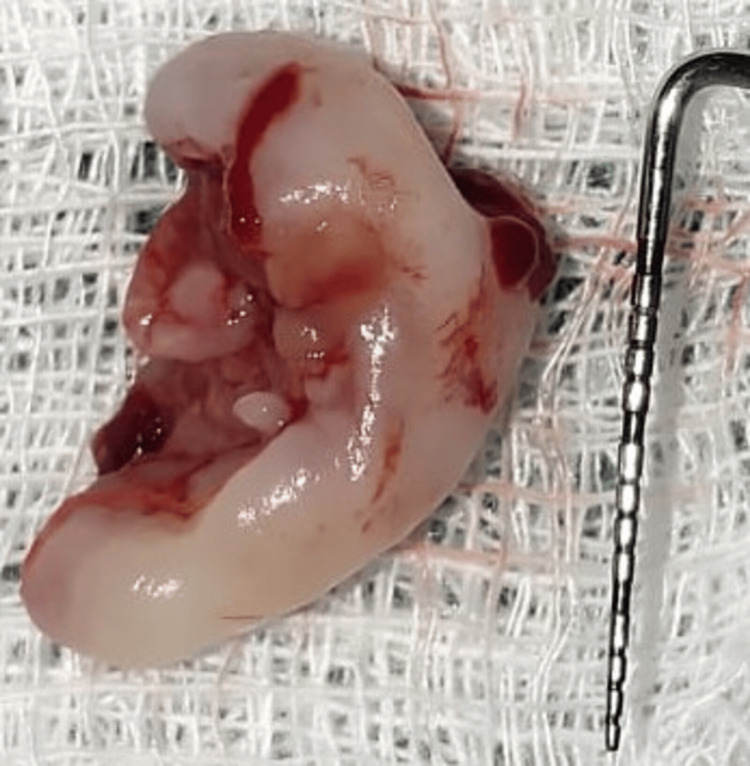
Excised tissue

The excised tissue (Figure 4) was then preserved in a formalin-filled container and given to the Department of Oral Pathology for histopathological examination of the tissue.

**Figure 5:**
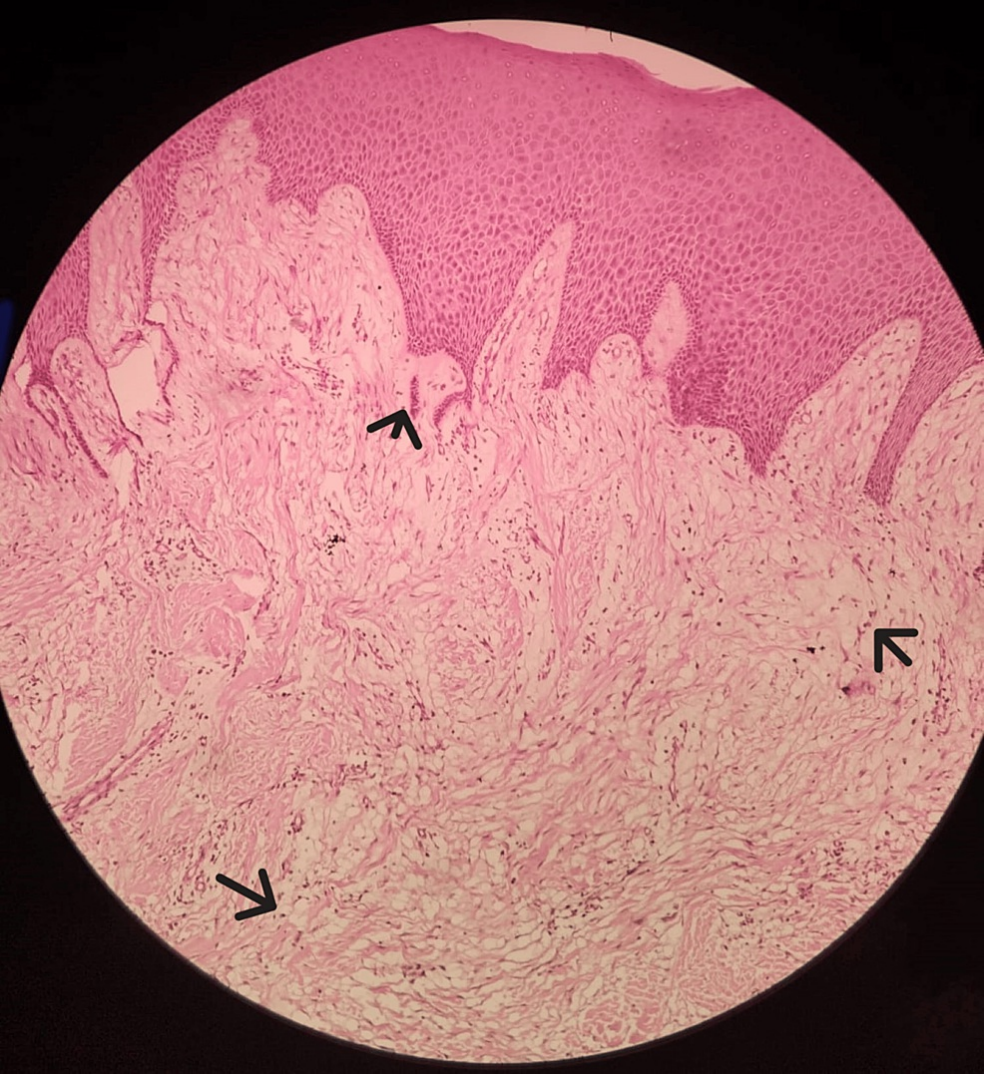
Histological view

Microscopically, the biopsy sample was composed of hyperplastic parakeratinized stratified squamous epithelium with long, thin, and anastomosing rete ridges, as well as fibrous connective tissue that contained numerous swollen fibroblasts (Figure 5). Therefore, histopathological diagnosis was given as fibroma.

**Figure 6:**
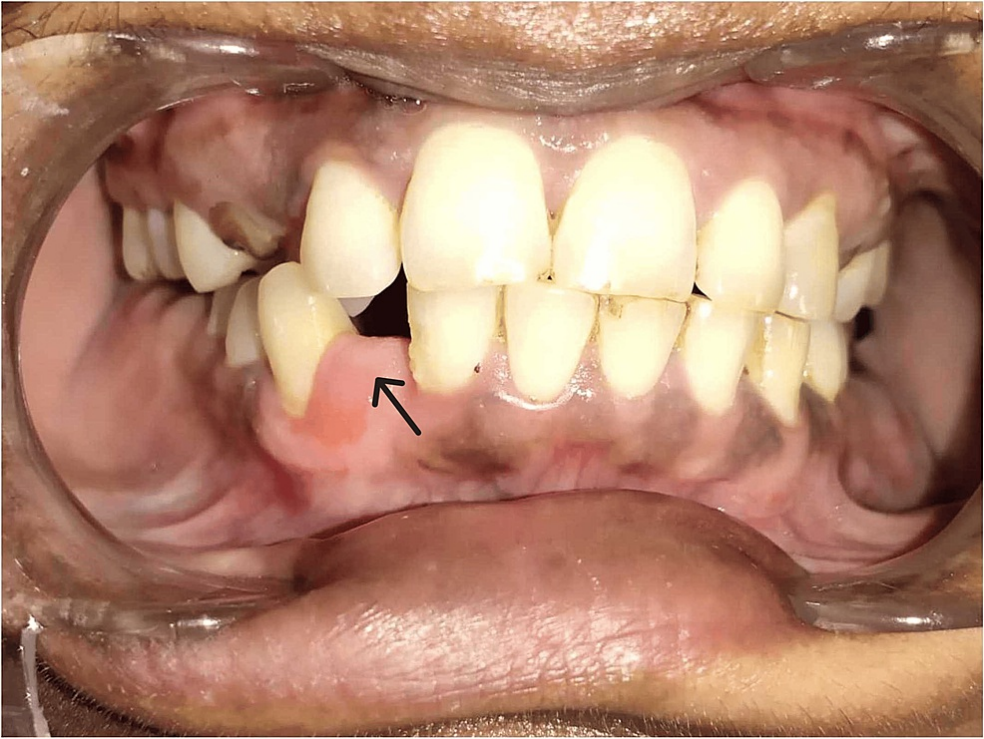
One month after operation

The patient was then recalled after one month for re-evaluation of the surgical site. Complete surgical site healing was seen after one month of the procedure (Figure 6).

## Discussion

Two categories of gingival disorders, plaque-induced and non-plaque-induced, include gingival overgrowth [6]; however, often, a more specific primary etiology can be identified [7]. There are different etiologies for gingival overgrowth. Etiologies of gingival overgrowth are inflammatory enlargement, drug-induced enlargement associated with systemic diseases, neoplastic enlargement, and false enlargement [7].

Inflammatory or traumatic gingival enlargement could be caused by the presence of sharp cusps, calculus, denture, or denture prosthesis [8]. It may be painful, and recurrences are often if there is the presence of overhanging margins of restorations, foreign bodies, chronic biting, and sharp cusp. Fibro-epithelial hyperplasia shows histological features, including fibrous connective tissue and hyperplastic parakeratinized stratified squamous epithelium. Clinically, it may or may not be symptomless [9].

Diode laser radiation can effectively, easily, and safely treat oral lesions. There is hardly any bleeding, little postoperative swelling, and few side effects from this operation. Better sight and less harm to nearby tissues are also benefits of laser irradiation. Laser surgery is more accurate, quicker, less painful, and causes less scarring and tissue contraction when treating soft tissue lesions. It also keeps the tissue's elastic properties intact [10].

Pai et al., in their case series, excision of gingival overgrowth using laser diode. Three cases of gingival overgrowth, which were diagnosed as fibroma, were described in 28, 45, and 36-year-old females [11]. Banerjee et al. described six cases of localized gingival overgrowth in patients between 35-50 years of age. Surgical excision of the tissues was carried out after full‑thickness mucoperiosteal flap elevation, followed by debridement. Histopathological diagnosis of the six cases were fibroma/ peripheral fibroma/ fibroepithelial polyp, pyogenic granuloma/ angiogranuloma, peripheral ossifying fibroma/ ossifying fibroma, peripheral fibroma with calcification, peripheral giant cell granuloma, peripheral ameloblastoma [12].

Total surgical removal and excision of the source are the preferable treatments for traumatic fibroma and all reactive hyperplasia [13]. Depending on clinical and structural factors, a variety of therapeutic modalities have been used for excision. The use of a traditional scalpel, electrosurgery, ablation with various infrared lasers, and even cryosurgery - a technique that uses liquid nitrogen and is very helpful in cases involving patients who are at high surgical risk, old, have coagulation issues, or are pacemaker carriers - are some of these options [14]. Ravindra et al., in their case report, gingival overgrowth removal assisted by laser in an endodontic perforation was done. The diagnosis of enlarged gingiva was given as unintentional perforation, resulting in gingival overgrowth during endodontic treatment [15].

The laser offers a lower risk of infection, sensitivity, and bleeding following the surgery because it is a less invasive procedure. It also implies low chances of postoperative complications such as bleeding and swelling. From the patients' perspective, most of them feel less anxious to have their dental health issues treated because laser therapy is gentle.

## Conclusions

Incorporating comfort, functionality, and aesthetics, modern dental care must lead to true oral health. Laser-assisted soft tissue treatment has several potential benefits. A laser can carry out the process, providing a fast, steady, bloodless, painless, and accurate healing experience. Modern treatment approaches, including laser-assisted soft tissue excision, combined with clinical and radiological examination, helped preserve the tooth and produce a stable and functional outcome.

## References

[1] Dannewitz B (2007). Proliferation of the gingiva: aetiology, risk factors and treatment modalities for gingival enlargement. Perio Pract Tod.

[2] Pal S, Hegde S, Ajil V (2012). The varying clinical presentations of peripheral ossifying fibroma: a report of three cases. Rev Odonto Cienc.

[3] Bouquot JE, Gundlach KK (1986). Oral exophytic lesions in 23,616 white Americans over 35 years of age. Oral Surg Oral Med Oral Pathol.

[4] Esmeili T, Lozada-Nur F, Epstein J (2005). Common benign oral soft tissue masses. Dent Clin North Am.

[5] Wood NK, Goaz PW (2006). Oral and maxillofacial lesions. Differential Diagnosis of Oral and Maxillofacial Lesions.

[6] Lindhe J, Lang NP, Karring T (2008). Clinical periodontology and implant dentistry. Blackwell Munksgaard.

[7] Armitage GC (1999). Development of a classification system for periodontal diseases and conditions. Ann Periodontol.

[8] Bagde H, Waghmare A, Savitha B, Vhanmane P (2013). Irritation fibroma - a case report. Int J Clin Dent.

[9] (2023). Peripheral ossifying fibroma. https://dermnetnz.org/topics/peripheral-ossifying-fibroma.

[10] Mandal A, Jalaluddin Jalaluddin, Sarkar S (2022). Diode laser assisted excision of gingival fibroma: a case report. Zenodo.

[11] Pai JB, Padma R, Divya Divya, Malagi S, Kamath V, Shridhar A, Mathews A (2014). Excision of fibroma with diode laser: a case series. J Dent Lasers.

[12] Banerjee S, Pal TK (2017). Localized gingival overgrowths: a report of six cases. Contemp Clin Dent.

[13] Rossmann JA (2011). Reactive lesions of the gingiva: diagnosis and treatment options. Open J Pathol.

[14] Ortega-Concepción D, Cano-Durán JA, Peña-Cardelles JF, Paredes-Rodríguez VM, González-Serrano J, López-Quilles J (2017). The application of diode laser in the treatment of oral soft tissues lesions: a literature review. J Clin Exp Dent.

[15] Ravindran DM, Sabarish R, Arul D, Ajit S, Harini DM (2019). Laser-assisted excision of gingival overgrowth in an endodontic perforation: a case report. Cureus.

